# Antibodies Related to *Borrelia burgdorferi* sensu lato, *Coxiella burnetii,* and *Francisella tularensis* Detected in Serum and Heart Rinses of Wild Small Mammals in the Czech Republic

**DOI:** 10.3390/pathogens10040419

**Published:** 2021-04-01

**Authors:** Alena Žákovská, Eva Bártová, Pavlína Pittermannová, Marie Budíková

**Affiliations:** 1 Department of Comparative Animal Physiology and General Zoology, Faculty of Science, Masaryk University, Kamenice 753/5, 625 00 Brno, Czech Republic; alenazak@sci.muni.cz; 2 Department of Biology, Faculty of Education, Masaryk University, Kamenice 753/5, 625 00 Brno, Czech Republic; 3 Department of Biology and Wildlife Diseases, Faculty of Veterinary Hygiene and Ecology, University of Veterinary and Pharmaceutical Sciences, Palackého tř. 1946/1, 612 42 Brno, Czech Republic; pittermannova.p@gmail.com; 4 Department of Mathematics and Statistics, Faculty of Science, Masaryk University, Kotlářská 2, 611 37 Brno, Czech Republic; budikova@math.muni.cz

**Keywords:** Lyme disease, Q fever, tularemia, zoonosis, rodents, seroprevalence

## Abstract

Wild small mammals are the most common reservoirs of pathogenic microorganisms that can cause zoonotic diseases. The aim of the study was to detect antibodies related to *Borrelia burgdorferi* sensu lato, *Coxiella burnetii,* and *Francisella tularensis* in wild small mammals from the Czech Republic. In total, sera or heart rinses of 211 wild small mammals (168 *Apodemus flavicollis*, 28 *Myodes glareolus*, 9 *A. sylvaticus*, and 6 *Sorex araneus*) were examined by modified enzyme-linked immunosorbent assay. Antibodies related to *B.* *burgdorferi* s.l., *C. burnetii*, and *F. tularensis* were detected in 15%, 19%, and 20% of animals, respectively. The prevalence of *B. burgdorferi* and *F. tularensis* statistically differed in localities and *F. tularensis* also differed in sex. Antibodies against 2–3 pathogens were found in 17% of animals with a higher prevalence in *M. glareolus*. This study brings new data about the prevalence of the above-mentioned pathogens.

## 1. Introduction

*Borrelia burgdorferi* s.s., *Borrelia afzelii*, and *Borrelia garinii* are responsible for causing Lyme disease (LD), a common zoonotic tick-borne disease in Europe [1]. About 32 vertebrate species were identified as reservoir hosts of *B. burgdorferi* s. l. Among them, small mammals and birds are the best-known host species, however deer, domestic animals (cats, dogs), and reptiles (lizards) can also serve as reservoirs [2]. Rodent species, for example, mice (*Apodemus*) and voles (*Clethrionomys*), have been studied as typical reservoir hosts of *B. burgdorferi* s.l. in various enzootic areas in Europe [3,4].

*Coxiella burnetii* can infect arthropods, birds, and mammals including humans and cause the zoonotic disease Q fever [5]. It is a bacterial pathogen that can be transmitted by ticks [6] with a wide range of hosts (cattle, sheep, goats, etc.) including small mammals [7]. Humans become infected mainly through the inhalation of aerosols or dust containing spore-like forms of *C. burnetii* [8].

*Francisella tularensis* is responsible for the zoonotic disease tularemia, which causes health problems especially in rabbits and hares [9]. However this microorganism was also isolated from different species of rodents [2]. Bacterium is transmitted by ticks, mosquitos, and fleas through direct contact with infected animals or by drinking contaminated water. Rodents represent an important reservoir of this pathogen [10]. The above-mentioned bacteria circulate in a sylvatic cycle between hosts, reservoirs, and vectors, with wild small mammals being important reservoirs.

In the Czech Republic there are around 4000 cases of Lyme disease in humans per year, one case of Q fever per year, and increasing cases of tularemia (in last ten years, 2011–2020, this number reached approximately 56 cases per year). According to these data, it is important to monitor the prevalence of above-mentioned infections in reservoirs. That is why the aim of this study was to detect antibodies related to *B. burgdorferi* s.l., *C. burnetii*, and *F. tularensis* in wild small mammals in order to find whether these pathogens are circulating in two frequently visited protected areas in the Czech Republic, and to identify the animal species that are the most sensitive to the above-mentioned infections.

## 2. Results

Antibodies related to *B. burgdorferi* s.l., *C. burnetii*, and *F. tularensis* were detected in 15%, 19%, and 20% of wild small mammals, respectively (Table 1). The prevalence of *B. burgdorferi* and *F. tularensis* differed according to locality (*p* = 0.0082 and *p* = 0.0254, respectively). The prevalence of *F. tularensis* also differed according to sex (*p* = 0.0298). There was no difference in the prevalence of all three infections in animal species (*p* > 0.05). However, the number of tested animals in the individual species was not comparable. In total, 27% of animals had antibodies related to at least to one infection. Antibodies related to 2–3 pathogens were found in 17% of animals, with higher prevalence of antibodies in *M. glareolus,* and with a statistical difference in sex (*p* = 0.022) and locality (*p* = 0.0169). A statistical difference was also found between the mutual positivity of *B. burgdorferi* and *C. burnetii* (*p* < 0.0001, odds ratio (OR) = 20.71, 95% CI for OR 8.34–51.44), *B. burgdorferi* and *F. tularensis* (*p* < 0.0001, OR = 30.86, 95% CI for OR 11.67–81.61), and *C. burnetii* and *F. tularensis* (*p* < 0.0001, OR = 56.89, 95% CI for OR 21.41–151.16).The values of OR for comparing the occurrence of antibodies against 2–3 pathogens in animals from the Moravian Karst and the Poodří was 8.13. The expected frequency of the occurrence of antibodies against 2–3 pathogens, assuming this occurrence to be independent from site or sex, was 30.2 for the Moravian Karst (with an observed frequency of 35) and 5.8 for the Poodří (with an observed frequency of 1). The occurrence of antibodies against 2–3 pathogens was 17.7 for females (with an observed frequency of 24) and 18.3 for males (with observed frequency 12).

The cut-off values for both IgG and IgM antibodies were higher in samples of heart rinses for all examined pathogens. The prevalence of both types of antibodies in positive samples was the same in both heart rinses and sera.

## 3. Discussion

The prevalence of *B. burgdorferi* s.l. (15%) was lower compared to the 44% prevalence determined by the same ELISA in heart rinses of wild small mammals in the same locality in North Moravia 10 years before [11]. Our results were similar in comparison to the 17–19% prevalence obtained by ELISA in wild small mammals in Slovakia, a country neighbouring the Czech Republic [12,13]. The animal species *A. flavicollis* was the main reservoir of infection, followed by *A. agrarius* and *M. glareolus.* In Poland, antibodies related to *B. burgdorferi* s.l. were detected by enzyme-labelled protein G assay in 58% of *M. glareolus* and 17% of *A. flavicollis* [14]. A much lower prevalence of *B. burgdorferi* s.l. antibodies (5% and 3.5%) was obtained by ELISA in *Apodemus* spp. and *M. glareolus,* respectively, in Italy [15].

Antibodies to *C. burnetii* were found in 19% of animals, without statistical difference between animal species. However, antibodies related to 2–3 pathogens were detected, with the highest prevalence in *M. glareous*. Similarly, Meredith et al. [16] detected antibodies related to *C. burnetii* in 17% of wild rodents from England and Scotland, also without a difference between animal species (16–19%) including *M. glareolus*, *A. sylvaticus*, and *Microtus agrestis.* It seems that animal species does not play an important role in the transmission of the above-mentioned infection. However, Pluta et al. [6] reported a 0% prevalence of *C. burnetii* in 119 tested rodents, including mainly *M. arvalis.* The animals were trapped in three Q fever endemic areas in Southern Germany. This is the reason why the authors suggested that this animal species did not play an essential role in the epidemiology of Q fever. In Italy, 143 rodents were trapped in 2008–2009 and tested by serological and molecular methods, with two *Apodemus* spp. found to be positive for *C. burnetii* (1.4%) [15]. In Austria, 110 animals including four animal species (*M. glareolus*, *A. flavicollis*, *A. sylvaticus*, and *M. arvalis*) were trapped in 2008 at two rural sites in Lower Austria and tested by serological methods and RT-PCR/PCR to detect *C. burnetii*, but with negative results [17].

Antibodies related to *F. tularensis* were found in in 20% of animals, without statistical difference between animal species. Christova and Gladinska [18] reported a 22% prevalence of *F. tularensis* in rodents in Bulgaria. However, this was in an area with the endemic occurrence of tularemia at the time. No antibodies related to *F. tularensis* were detected by ELISA in 143 wild rodents from Italy [15]. Outside Europe, a low prevalence of *F. tularensis* has been detected, e.g., 5% in Iran [19], 4.8% in China [20], and 0.8–1.7% in Japan [3].

The prevalence of *B. burgdorferi* and *F. tularensis* antibodies was statistically higher in the Moravian Karst compared to Poodří. The same was also found in the case of *C. burnetii,* but without a statistical difference. This could be explained by the fact that the Moravian Karst is localized in South Moravia, near agricultural land, compared to Poodří, which is situated in the north near high mountains (Jeseníky and Beskydy) that are less populated. Locality is thus another important factor that contributes to the maintenance and spread of infection. Poodří is located adjacent to southern Poland, where antibodies to *C. burnetii* and *F. tularensis* were detected in 6.5% and 3.2%, respectively, of 216 employees of National Forests [4], meaning that these infections are circulating in that area.

A higher prevalence of *F. tularensis* antibodies was found in females compared to males. In addition, antibodies to 2–3 pathogens were detected more frequently in females. Sunagar et al. [21] described the impact of gender on vaccine efficacy against tularemia infection in C57BL/6Tac mice. They found enhanced levels of *Ft*-specific Abs in serum and broncho-alveolar lavage fluid post-challenge, improving the survival of i*Ft*-vaccinated females compared to males. These results could emphasise the fact that gender differences must be a serious consideration in tularemia or vaccine development studies.

Statistical difference was found between the mutual positivity of *B. burgdorferi* and *C. burnetii*, *B. burgdorferi* and *F. tularensis,* and *C. burnetii* and *F. tularensis.* This fact may indicate that some pathogen binding could be influenced by various factors such as type of hosts or their defence mechanisms, raising a question which requires further study. We also found three animal species (*A. flavicolis, M. glareous,* and *S. araneus*) to have antibodies against three pathogens *B. burgdorferi* s.l., *C. burnetii*, and *F. tularensis*, at a prevalence of 16.7–25%. To our knowledge, there is no information on the presence of antibodies related to 2–3 pathogens in these animal species. A relatively high prevalence of *B. burgdorferi* s.l. and *F. tularensis* with coinfection in different species of rodents was detected by PCR in small mammals, as well as in ticks collected in the Austrian and Slovakian borderland (a region endemic for tularemia), neighbouring countries of the Czech Republic [22]. The authors suggested that the prevalence of borreliosis could be modified in different animal species during outbreaks of tularemia. Other coinfections (*B. burgdorferi* s.l. and *F. tularensis*) were detected in ticks in Germany [23], and the coinfection of *C. burnetii* and *F. tularensis* was detected by real-time PCR in wild animals and ticks in Poland [24].

Wild small mammals (rodents) can participate in the circulation and maintenance of the pathogenic microorganisms in a sylvatic cycle, thus risking the development and focal spreading of infectious diseases. Antibodies related to *C. burnetii* were detected, e.g., in 41% of foxes from the United Kingdom [16], and antibodies related to *F. tularensis* were detected in 7% of foxes from Germany [25]. Small mammals, mainly rodents, represent the basis of food for carnivores and thus could be the main source of these infections.

More than 75% of human diseases are of zoonotic origin and are related to contact with wildlife and domestic animals [26]. For example, antibodies related to *C. burnetii* were detected in 6.5% of employees of the National Forests in Poland [4] and in 39% of 151 Polish farm workers [27]. The frequency of contact between wildlife and humans in urban and peri-urban areas has changed over time from sporadic encounters to the permanent sharing of the environment, thus the chance of parasitic transmission to humans and domestic animals is increasing [28]. The same animal species of rodents as those in our study were caught inside human dwellings and outside in nature in Senegal and examined for thirteen pathogens by PCR [29]. Based on the results, the authors concluded that the presence of rodents in human dwellings can pose a significant risk of transmission of pathogens to domestic animals and humans.

This study showed the circulation of the above-mentioned bacteria in rodent populations and their possible involvement as hosts in the sylvatic cycle. It is important to continue the monitoring of these infections, because, while clinical cases of Q fever occur very rarely, the number of human patients suffering from tularemia and Lyme disease is gradually increasing, affecting on average 0.56 and 40.1 per 100,000 inhabitants of the Czech Republic in the last 10 years, respectively (webpage of State Health Institute).

## 4. Material and Methods

Sampling was done in two localities in the Czech Republic. The first locality was the protected landscape of the Moravian Karst (South Moravia, GPS: 49°21′49.439″ N, 16°42′28.210″ E), with trapping carried out along the Punkva River. The locality is characterized by beech forests with preserved species composition, complemented by oak and hornbeam forests and some wet meadows along the river. The second locality was the protected landscape of Poodří (North Moravia, GPS: 49°43′26.360″ N, 18°6′27.415″ E), with trapping carried out around the Bažantula pond and the area close to the town of Studénka. This locality is characterized by an oak Ficario-Ulmetum alnetosum association forests, alternating with meadows.

Wild small mammals were trapped with snapping traps and “life-hunt” traps in 2014 (spring and autumn), according to approved experimental procedures. Traps were placed on the ground in a line, seven metres apart, and checked the next morning. In total, 211 wild small mammals, including 168 yellow-necked mice (*Apodemus flavicollis*), 28 bank voles (*Myodes glareolus*), nine long-tailed field mice (*A. sylvaticus*), and six Eurasian shrews (*Sorex araneus*) were trapped. Data about the animals and localities (animal species, sex, and locality) are summarized in Table 1. Living animals (*n* = 108) were anesthetized to obtain blood from the neck artery. Blood was centrifuged to obtain serum that was stored at −18 °C. Animals trapped by snapping springs (*n* = 103) were dissected and the hearts of individual animals were put into a 0.85% physiological solution for 1–2 days at 4 °C. After the removal of the heart, the solution was centrifuged and the drained supernatant (heart rinse) was stored at −18 °C until assay.

Samples (108 sera and 103 heart rinses) were examined using modified enzyme-linked immunosorbent assay (ELISA, TestLine, Brno, Czech Republic), according to the procedure described by Vostal and Žákovská [11], in the following manner: microplates were parallelly filled with 100 μL of the respective antigen diluted in a carbonate buffer at pH 9.6 (2 μg/mL) and incubated overnight at 4 °C. After washing 3 times with a phosphate buffer (pH 7.4) containing 0.05% Tween 20, 100 μL of sera (diluted at 1:100 in the phosphate buffer with 0.05% Tween 20 and 0.3% casein to the mean serum protein concentration 0.68 mg/mL) or heart-rinses (diluted to the same protein concentration) were added and incubated at 37 °C for 1 h. After a triple washing of the plates, 100 μL of goat anti-mouse IgM and IgG peroxidase conjugates (Sigma-Aldrich spol. s.r.o., Prague, Czech Republic) were added per well. After 1 hour of incubation and a subsequent washing, 100 μL per well of substrate solution (0.1 M citrate buffer pH 4.7–5.0 with 0.05% H_2_O_2_) with orthophenylene diamine were added. The reaction was stopped with 1 M H_2_SO_4_ after 10–20 min of incubation. The absorbance of the samples was measured at 492 nm by spectrophotometer (SLT RainBow, Schoeller instruments, s.r.o., Prague, Czech Republic). Positive controls were prepared by immunization of BALB/c mice with 300 μL of a mixture of ultrasonically disrupted whole cell antigens of *B. afzelii* BRZX27 MSLB 8065, *B. garinii* BRZX 23 MSLB 8064, *B. burgdorferi* s.s. WSLB 8014/1 (Bioveta a.s., Ivanovice na Hané, Czech Republic), *F. tularensis* (Bioveta, a.s., Ivanovice na Hané, Czech Republic), or *C. burnetii* (Batch NO. 87, Virological Institute Academy of Sciences, Bratislava, Slovakia) in the dose of 40 μg/mL of antigen and one mg/mL aluminium hydroxide in 0.85% physiological solution, according to Žákovská et al. [30]. The same but inactivated suspensions of *B. burgdorferi* s.l., *F. tularensis*, and *C. burnetii* were used as antigens in ELISA. The serum of wild mice negative to *B. burgdorferi* s.l., *F. tularensis,* and *C. burnetii* antigen was used as negative control. Cluster analysis with the K-diameter method was used for the evaluation of IgM and IgG ELISA positive, dubious, and negative samples. The cluster analysis was applied to the data plotted in the sub-graphs. The data showed normal distribution based on the Shapiro–Wilk and Kolmogorov–Smirnov tests [31]. According to the results of the cluster analysis, the absorbance of IgM antibodies for negative samples ranged between 0–0.1, the cut-off value was 1.0–1.5, and the samples were marked as positive where they had values above 1.5. The absorbance of IgG antibodies for negative samples ranged between 0–0.1, the cut-off value was 0.1–0.3, and the samples were marked as positive where they had values above 0.3.

The data analysis was performed with the Pearson Chi-square test for independence, using STATISTICA Cz 12 [31] or by the Monte Carlo method using IBM SPSS Statistics 20. We tested the null hypothesis that seroprevalence of bacteria does not differ in animal species, sex, and locality. The differences were considered statistically significant if the *p*-value was ≤0.05. In the case of the rejection of the null hypothesis, Scheffe’s multiple comparison method [31] was used. The probability of the occurrence of antibodies in two different groups (two different localities, male and female) was set by calculating the odds ratio (OR). If the OR was close to 1, it meant that there was no difference in the occurrence of the observed characteristic in the two observed groups.

## Figures and Tables

**Table 1:** The results of the serological examination (*Borrelia burgdorferi* s.l., *Coxiella burnetii*, and *Francisela tularensis*) of wild small mammals according to different characteristics (species, sex, and locality) captured in the Czech Republic (2014).

Characteristics	Animals Tested	Positive (%)				
		*B. burgdorferi*	*C. burnetii*	*F. tularensis*	At Least One Infection	Antibodies to 2–3 Pathogens
**Species**						
*Apodemus flavicollis*	168	25 (15%)	34 (20%)	34 (20%)	47 (28%)	28 (17%)
*Apodemus sylvaticus*	9	0 (0%)	0 (0%)	0 (0%)	0 (0%)	0 (0%)
*Myodes glareous*	28	4 (14%)	6 (21%)	7 (25%)	8 (29%)	7 (25%)
*Sorex araneus*	6	2 (33%)	1 (17%)	1 (17%)	2 (33%)	1 (17%)
Statistical significance		*p* > 0.05	*p* > 0.05	*p* > 0.05	*p* > 0.05	*p* > 0.05
**Sex**						
Female	104	19 (18%)	25 (24%)	27 (26%)	33 (32%)	24 (23%)
Male	107	12 (11%)	16 (15%)	15 (14%)	24 (22%)	12 (11%)
Statistical significance		*p* > 0.05	*p* > 0.05	*p* = 0.0298 *	*p* > 0.05	*p* = 0.022 *
Power of the test				1 − β = 0.59		1 − β = 0.63
Odds ratio, 95% CI for OR				OR = 2.15, 1.1–4.3		OR = 2.38, 1.12–5.05
**Locality**						
Moravian Karst	177	31 (18%)	37 (20%)	40 (23%)	52 (29%)	35 (20%)
Poodří	34	0 (0%)	4 (12%)	2 (6%)	5 (15%)	1 (3%)
Statistical significance		*p* = 0.0082 *	*p* > 0.05	*p* = 0.0254 *	*p* > 0.05	*p* = 0.0169 *
Power of the test		1 − β = 0.94		1 − β = 0.68	1 − β = 1	1 − β = 0.77
Odds ratio, 95% CI for OR		NT		OR = 4.67, 1.1–20.3	OR = 10.31, 4.3–26.7	OR = 8.13, 1.08–61.5
**Total**	211	31 (15%)	41 (19%)	42 (20%)	57 (27%)	36 (17%)

* Statistical difference *p* < 0.05. OR: odds ratio; CI: confidence interval; NT = not tested.

## Data Availability

Data is contained within the article.
